# Supplementary material for: Marcksl1 modulates endothelial cell mechanoresponse to haemodynamic forces to control blood vessel shape and size
Source: Nat Commun. 2020 Oct 30;11:5476. doi: 10.1038/s41467-020-19308-5 (PMC7603353; doi:10.1038/s41467-020-19308-5)
Supplement: Supplementary file 1 — Supplementary Information [file 41467_2020_19308_MOESM1_ESM.pdf]

Supplementary Information

Marcksl1 modulates endothelial cell mechanoresponse to haemodynamic forces to control blood vessel shape and size.

Igor Kondrychyn, Douglas J. Kelly, Núria Taberner Carretero, Akane Nomori, Kagayaki Kato, Jeronica Chong, Hiroyuki Nakajima, Satoru Okuda, Naoki Mochizuki and Li-Kun Phng

This file includes:

Supplementary Figures 1-13
Supplementary Tables 1-2
Supplementary reference

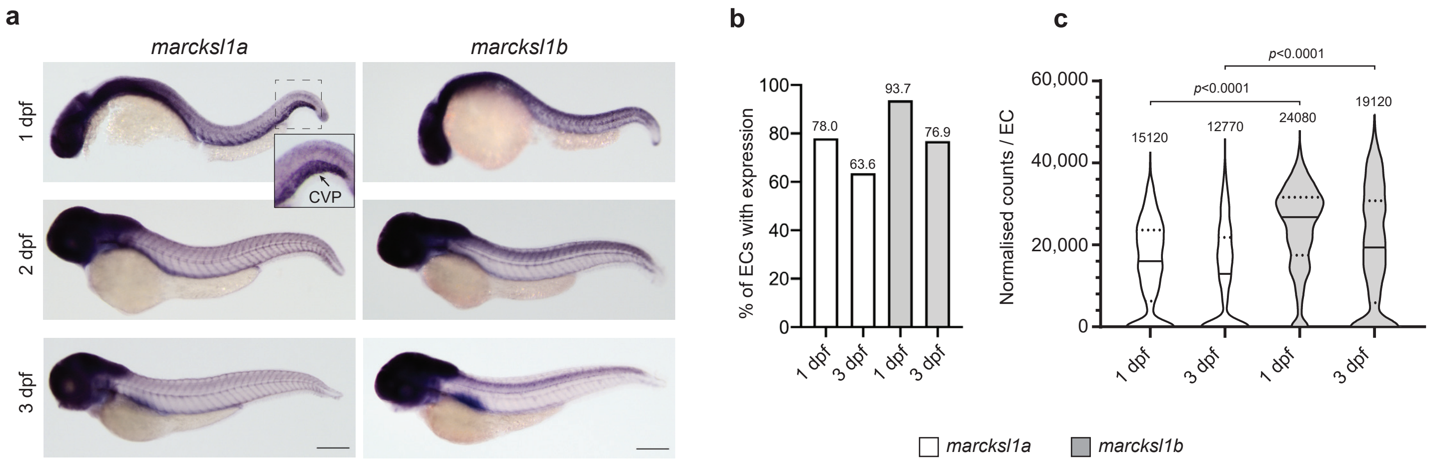

Supplementary Figure 1. *marcksl1a* and *marcksl1b* mRNA expression. **a** *In situ* hybridization with *marcksl1a* and *marcksl1b* RNA probes at different developmental stages. The inset shows a magnified view of the caudal vein plexus (CVP). Images are representative of 10 embryos for every stage and probe (n=2 independent experiments). **b, c** scRNAseq analysis showing *marcksl1a* and *marcksl1b* expression levels in endothelial cells (ECs) of 1 and 3 dpf embryos (1 dpf: *n*=969 cells; 3 dpf: *n*=1074 cells; 3 independent experiments). Data was analysed by ordinary one-way ANOVA with Tukey's multiple comparison test (**c**). Violin plots represent the entire range of values, dotted lines indicate first and third quartiles, center line is median. Mean values are indicated. Scale bar, 200 μm. Source data are provided as a Source Data file.

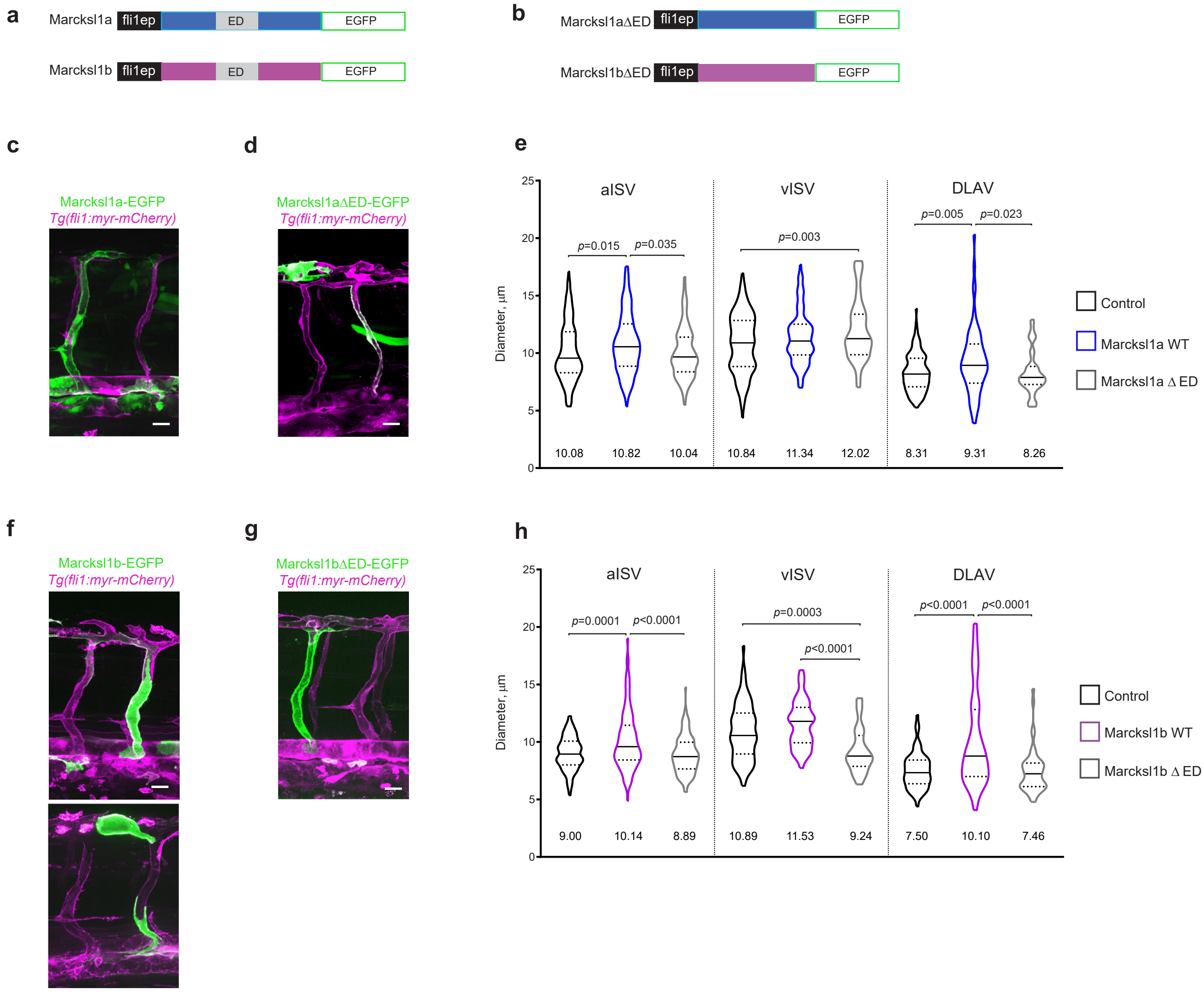

Supplementary Figure 2. Marcksl1 expression level regulates blood vessel diameter. Plasmid constructs encoding *fli1ep*-driven expression of full-length (**a**) or mutated (**b**, without an Effector domain, ED) Marcksl1a or Marcksl1b proteins tagged with EGFP. Maximum intensity projection of a confocal z-stack of ISVs in zebrafish trunk of 2 dpf *Tg(fli1:myr-mCherry)^{ncv1}* transgenic embryos expressing full length Marcksl1a (**c**), Marcksl1aΔED (**d**), full length Marck-sl1b (**f**) or Marcksl1bΔED (**g**). Marcksl1 overexpressing cells are in green. **e** Quantification of aISV, vISV and DLAV diameter in control and Marcksl1a-over-expressing blood vessels (control: *n*=27 aISVs/30 vISVs/24 DLAVs from 34 embryos; full length Marcksl1a: *n*=25 aISVs/18 vISVs/13 DLAVs from 20 embryos; Marcksl1aΔED: *n*=17 aISVs/13 vISVs/9 DLAVs from 14 embryos). **h** Quantification of aISV, vISV and DLAV diameter in control and Marck-sl1b-overexpressing vessels (control: *n*=17 aISVs/15 vISVs/21 DLAVs from 38 embryos; full length Marcksl1b: *n*=21 aISVs/18 vISVs/22 DLAVs from 23 embryos; Marcksl1aΔED: *n*=25 aISVs/10 vISVs/14 DLAVs from 15 embryos). Violin plots represent the entire range of values, dotted lines indicate first and third quartiles, center line is median. Mean values are indicated. Statistical significance was determined by ordinary one-way ANOVA with Tukey's multiple comparisons test. DLAV, dorsal longitudinal anastomotic vessel; ISV, intersegmental vessel; aISV, arterial ISV; vISV, venous ISV. Scale bars, 20 µm. Source data are provided as a Source Data file.

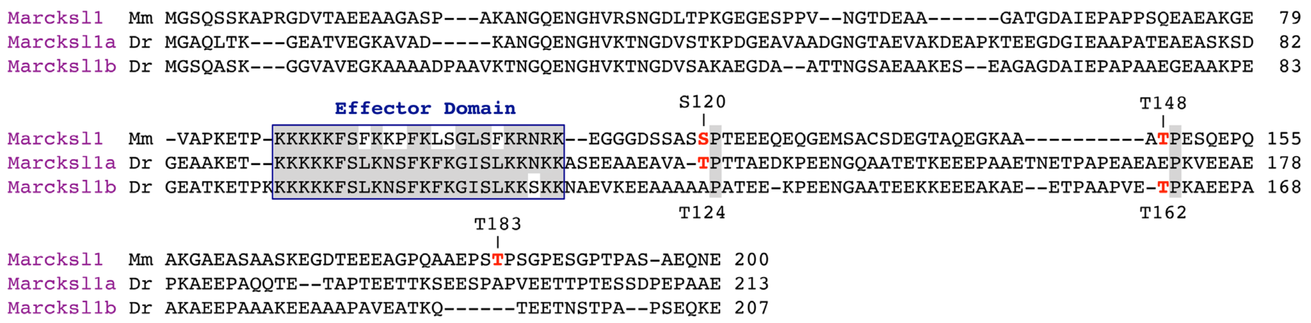

Supplementary Figure 3. Mouse and zebrafish Marcksl1 proteins share sequence homology. The conserved positively charged Effector Domain and putative phosphorylation sites (in red), T124 in Marcksl1a and T162 in Marcksl1b, are shown. T124 and T162 sites correspond to experimentally validated (ref. 1) JNK kinase phosphorylation sites, S120 and T148, respectively, in mouse Marcksl1. Dr, *Danio rerio*; Mm, *Mus musculus*.

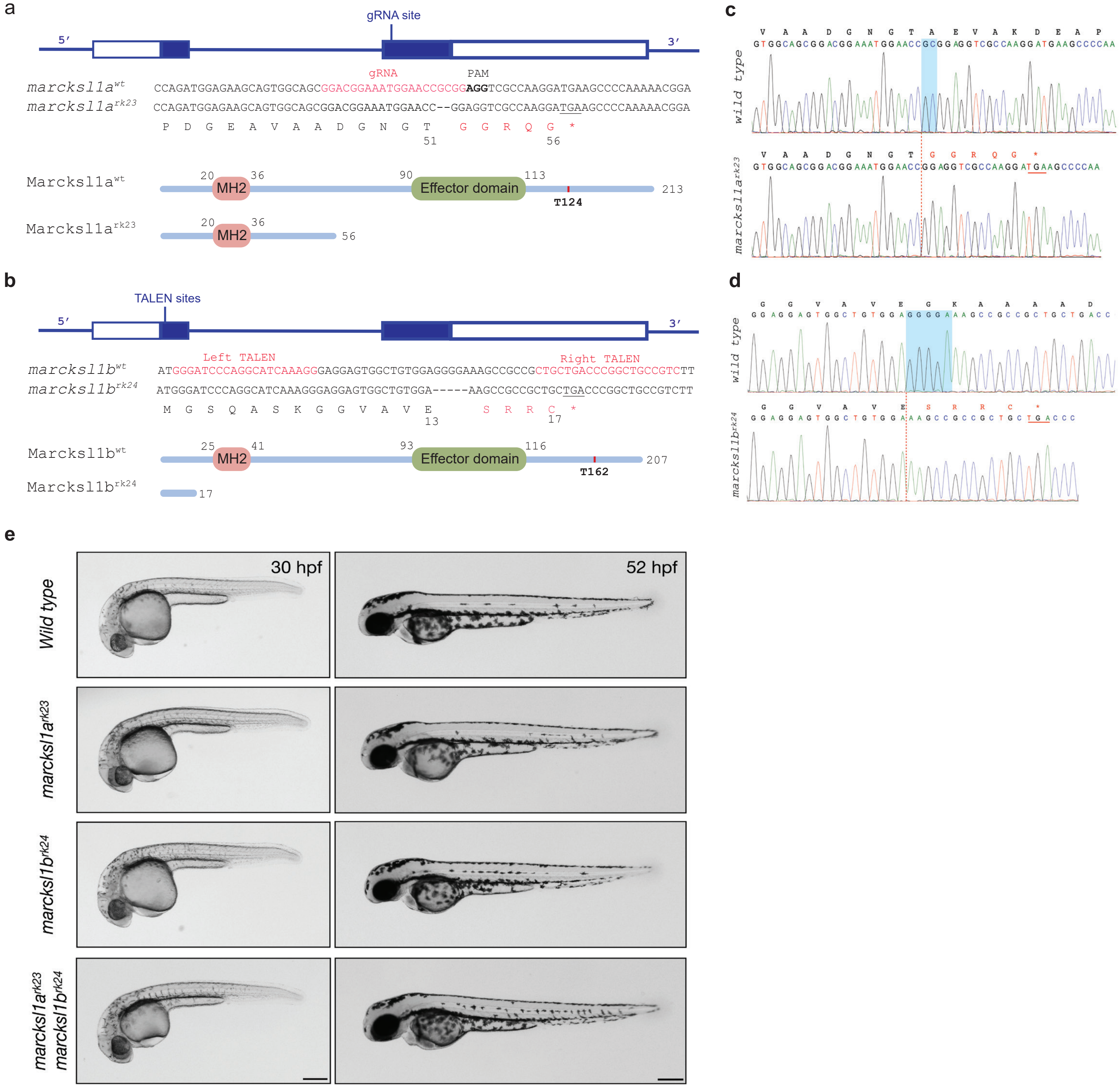

Supplementary Figure 4. Generation of *marcksl1a* and *marcksl1b* mutant zebrafish. **a**, **b** CRISPR/Cas9- and TALEN-mediated mutagenesis of *marcksl1a* and *marcksl1b* genes, respectively. **a** Zebrafish *marcksl1a* gene structure, gRNA binding site (in red), $marcksl1a^{rk23}$ mutant allele, Marcksl1a wild type (213 aa) and $Marcksl1a^{rk23}$ (truncated at 56 aa) protein structure. The *rk23* mutation causes a 2-nt deletion which leads to a frameshift after 51 aa and premature stop codon (underlined), and as a result to the loss of the Effector Domain and JNK phosphorylation site, T124. **b** Zebrafish *marcksl1b* gene structure, a pair of TALEN binding sites (in red), $marcksl1b^{rk24}$ mutant allele, Marcksl1b wild type (207 aa) and $Marcksl1b^{rk24}$ (truncated at 17 aa) protein structure. The *rk24* mutation causes a 5-nt deletion which leads to a frameshift after 13 aa and premature stop codon (underlined), and as a result to loss of MH2 and Effector domains and JNK phosphorylation site, T162. Non-coding and coding parts of exons are shown as white and dark blue rectangles, respectively. The partial nucleotide and amino acid sequences are shown. **c**, **d** Sequence reads showing 2-nt and 5-nt deletions (shadowed area in wild type) in $marcksl1a^{rk23}$ (**c**) and $marcksl1b^{rk24}$ (**d**) alleles (red dotted line), respectively. Premature stop codons are underlined. Amino acid sequences are shown above the nucleotide sequences. **e** Bright field images of wild type, $marcksl1a^{rk23}$, $marcksl1b^{rk24}$ and $marcksl1a^{rk23}$;$marcksl1b^{rk24}$ mutants at 30 and 52 hpf. Images are representa-tive of 10 embryos from every stage and genotype. Scale bar, 250 μm.

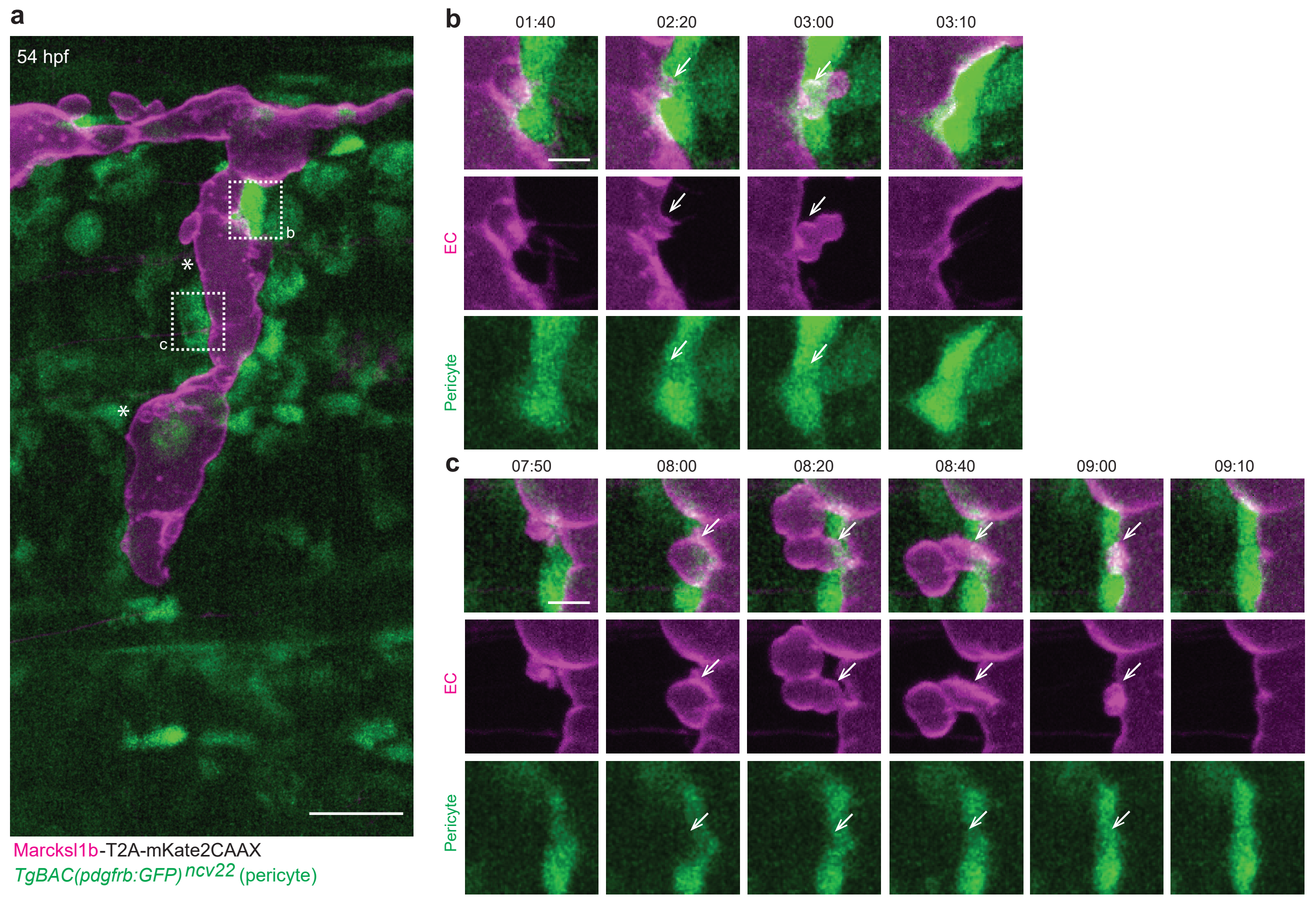

Supplementary Figure 5. Marcksl1-induced increase in vessel diameter and membrane blebbing occur in the presence of pericytes. **a–c** Maximum intensity projection of a confocal z-stack of an ISV and DLAV of *TgBAC(pdgfrb:GFP)*^{ncv22} with mosaic expression of Marcksl1b-T2A-mKate2CAAX in ECs (magenta) at 54 hpf. Pericytes are labelled green. Asterisks, regions of vessel that are wider. **b, c** Still images of a time-lapse movie. Arrow, basal blebs protrude in areas of blood vessel wrapped by pericytes. Representative movie of 4 movies (n=4 embryos, 2 independent experiments). 00:00, hh:mm. EC, endothelial cell. Scale bars, 20 μm (**a**) and 5 μm (**b**, **c**).

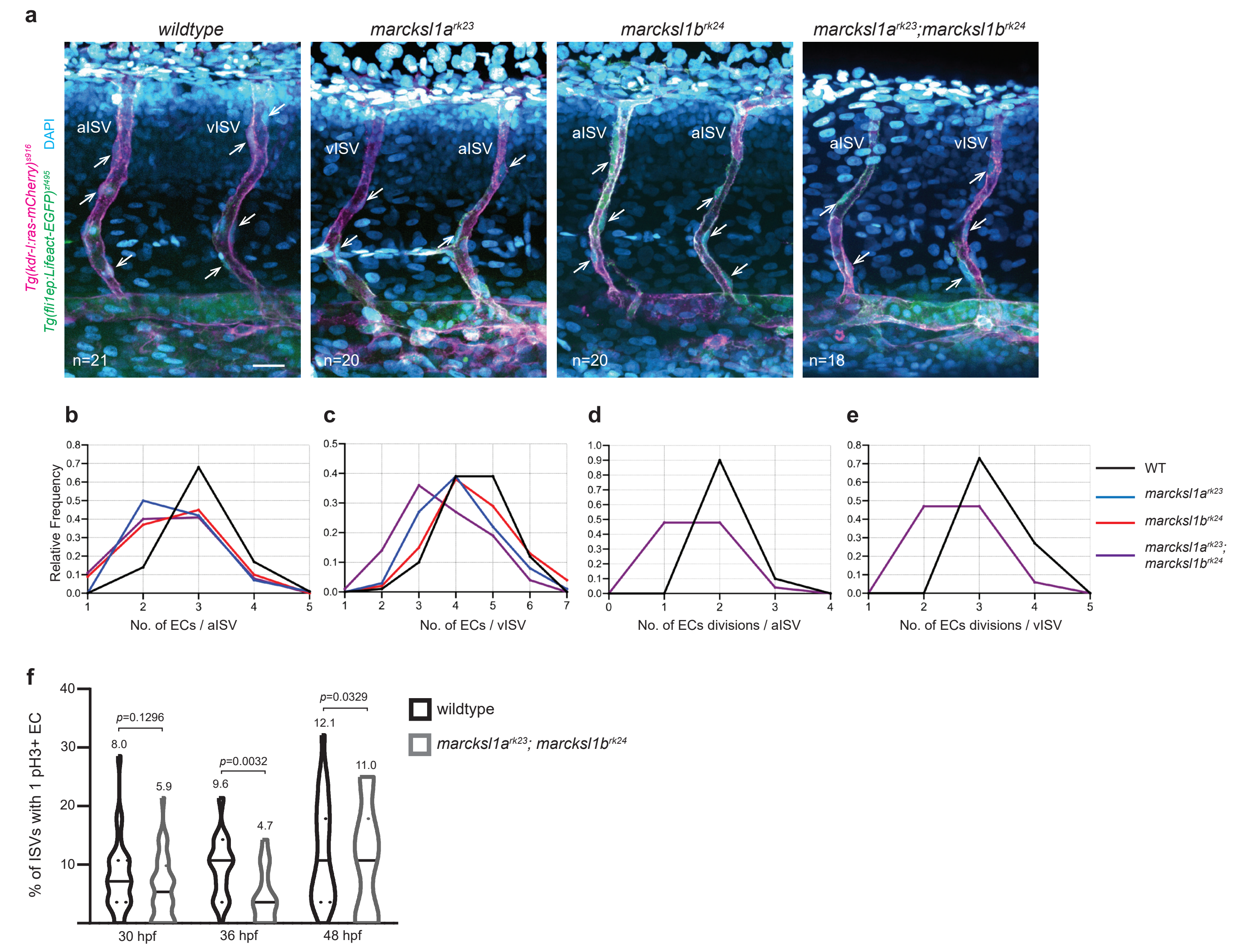

Supplementary Figure 6. Marcksl1 promotes endothelial cell proliferation. **a** Maximum intensity projection of confocal z-stacks of ISVs of 52 hpf wildtype and *marcksl1* mutant embryos in *Tg(fli1ep:Lifeact-EGFP)^{zf495};Tg(kdr-l:ras-mCherry)^{s916}* background. Cell nuclei are detected by DAPI staining. Arrows indicate endothelial nuclei in ISVs; *n* shows number of the examined embryos. **b-c** Quantification of EC number in arterial (**b**) and venous (**c**) ISVs at 52 hpf. Graphs show the relative frequency of nucleus number found in each ISV (wildtype: *n*=139 aISVs/111 vISVs from 21 embryos; *marcksl1a^{rk23}*: *n*=121 aISVs/163 vISVs from 20 embryos; *marcksl1b^{rk24}*: *n*=156 aISVs/124 vISVs from 20 embryos; *marcksl1a^{rk23};marcksl1b^{rk24}*: *n*=167 aISVs/86 vISVs from 18 embryos). Data are collected from 4 independent experiments. **d-e** Quantification of EC divisions in arterial (**d**) and venous (**e**) ISVs from time-lapse movies from 24 hpf to 48 hpf. Graphs show the relative frequency of cell divisions found in ISVs (wildtype: *n*=31 aISVs/11 vISVs from 11 embryos; *marcksl1a^{rk23};marcksl1b^{rk24}*: *n*=23 aISVs/17 vISVs from 9 embryos). Data are collected from 3 independent experiments. **f** Quantification of ISVs with one pH3 positive EC in wildtype and *marcksl1a^{rk23};marcksl1b^{rk24}* embryos at 30 hpf (*n*=30 wildtype/30 mutant embryos), 36 hpf (*n*=30 wildtype/26 mutant embryos) and 48 hpf (*n*=23 wildtype/32 mutant embryos). Data collected from 3 independent experiments. Violin plots represent the entire range of values, dotted lines indicate first and third quartiles, center line is median. Mean values are indicated. Statistical significance was determined by two-tailed unpaired *t*-test. EC, endothelial cell; ISV, intersegmental vessel; aISV, arterial ISV; vISV, venous ISV. Scale bar, 20 μm. Source data are provided as a Source Data file.

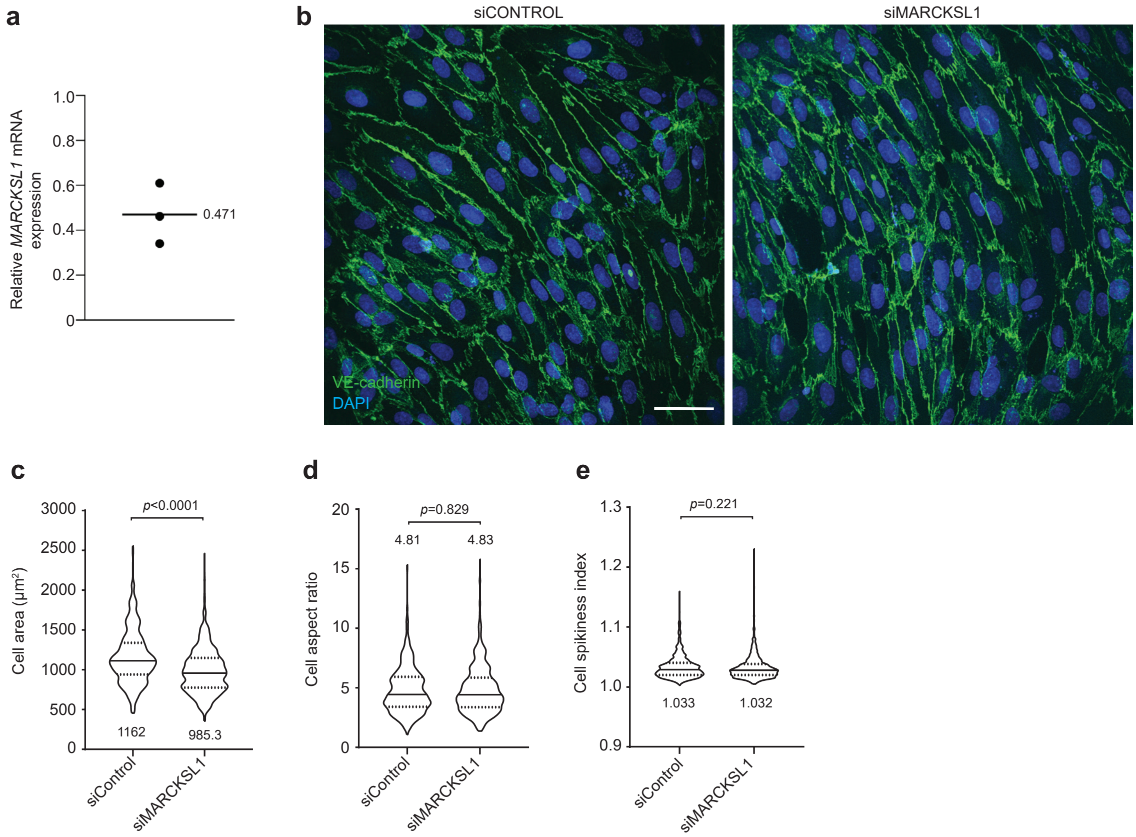

Supplementary Figure 7. Knockdown of MARCKSL1 decreases HUVEC size. **a** *MARCKSL1* mRNA expression level after siRNA knockdown (n=3 independent experiments). **b** Maximum intensity projection of confocal z-stacks of HUVECs transfected with control non-targeting siRNA or MARCKSL1 siRNA 2 days post-transfection. **c–e** Quantification of cell area (**c**), cell spikiness index (**d**) and cell aspect ratio (**e**; siCONTROL: n=233 cells; siMARCKSL1: n=431 cells from 3 independent experiments). Statistical significance was assessed by two-tailed unpaired t-test. Violin plots represent the entire range of values, dotted lines indicate first and third quartiles, center line is median. Mean values are indicated. Scale bars, 50 μm. Source data are provided as a Source Data file.

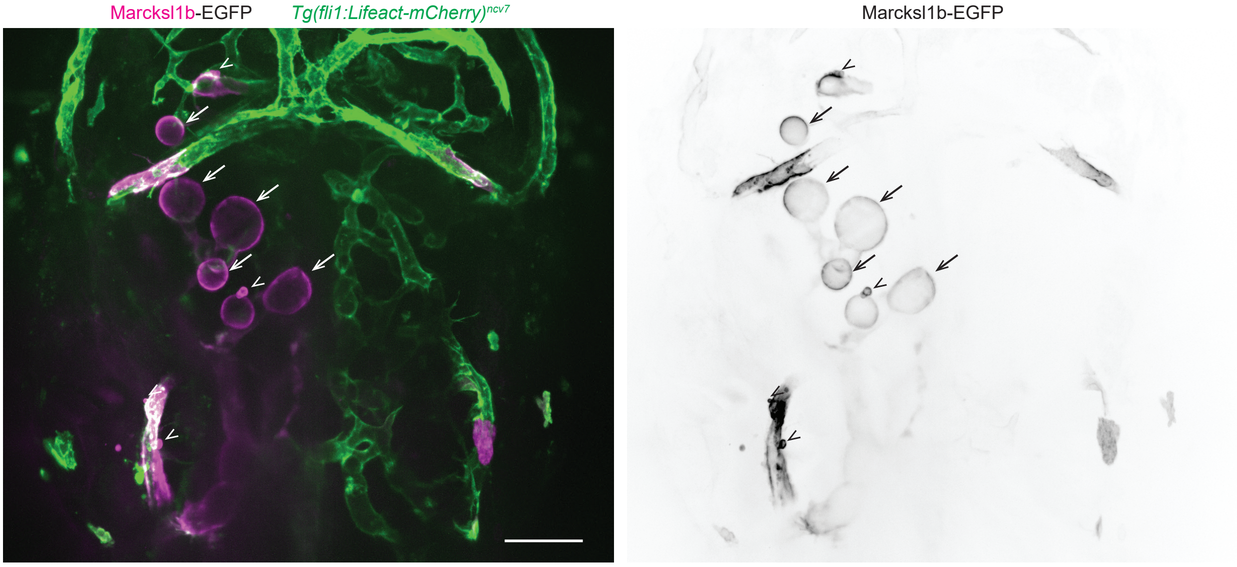

Supplementary Figure 8. Marcksl1 induces ectopic blebbing in hindbrain vessels. Maximum intensity projection of a confocal z-stack of hindbrain vessels of 72 hpf *Tg(fli1:Lifeact-mCherry)*^{ncv7} embryos with mosaic expression of Marcksl1b-EGFP in endothelial cells. Arrows, local dilation of blood vessels. Arrowheads, blebs. Image is representative of 4 embryos over 2 independent experiments. Scale bar, 50 µm.

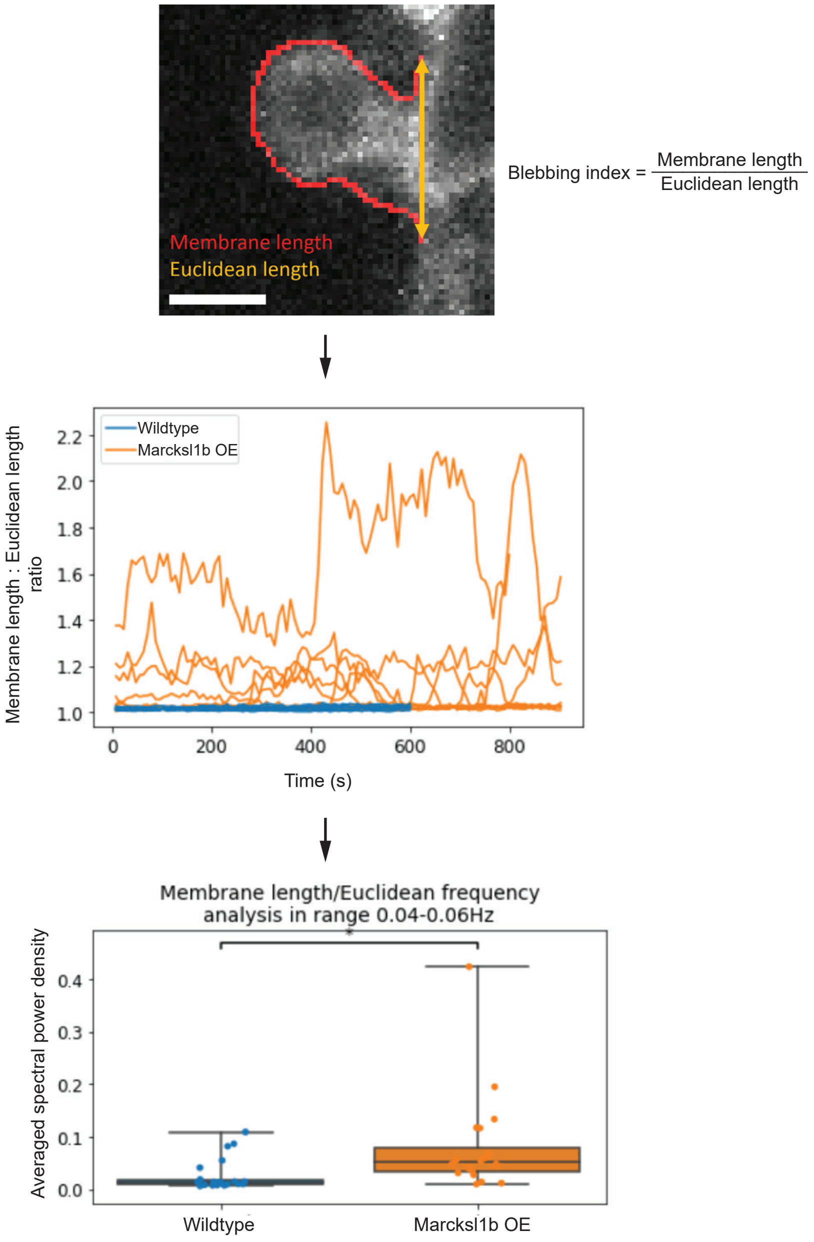

Supplementary Figure 9. Methodology to quantify membrane blebbing. Membrane blebbing index is measured as a ratio between membrane length of the bleb and Euclidean length at each timepoint of a times series. The spectral power density was then obtained and averaged over a window of interest (0.04 – 0.06 Hz) during which blebs form. The average spectral power density for wildtype and Marcksl1-overexpressing (OE) endothelial cells was plotted (wildtype: n=7 embryos; Marcksl1b OE: n=7 embryos; 4 independent experiments). Box plot shows median (horizontal line), boxes represent the first and third quartile, whiskers show standard deviation, individual points are shown. Statistics was evaluated by two-tailed unpaired *t* test with Welch's correction, *p=0.0229. Scale bar, 2 μm.

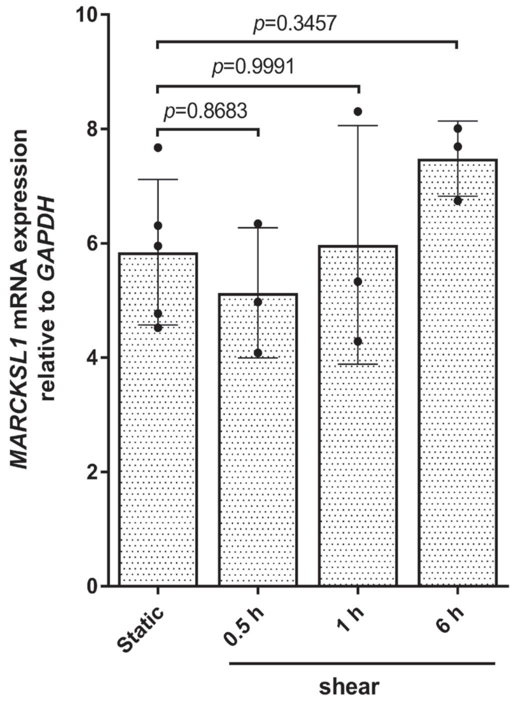

Supplementary Figure 10. *MARCKSL1* mRNA expression is not regulated by shear stress *in vitro*. HPAECs were exposed to static or 15 dyn/cm^{-2} laminar shear stress for 0.5, 1 or 6 hours. *MARCKSL1* mRNA expression is shown relative to *GAPDH* expression. Data are mean +/- s.d. of 3 independent experiments. Statistical analysis was performed by one-way ANOVA and Sidak's multiple comparison test. Source data are provided as a Source Data file.

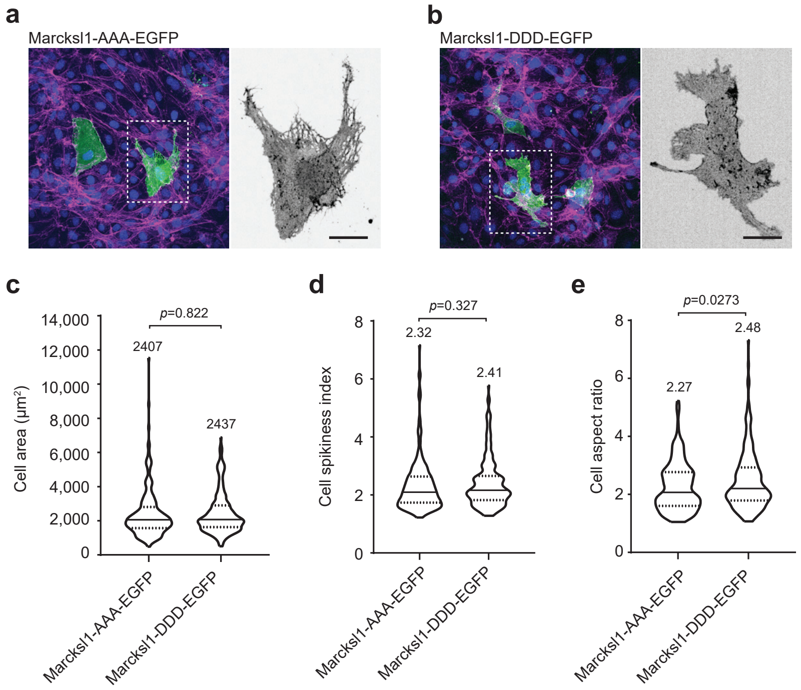

Supplementary Figure 11. JNK phosphorylation of Marcksl1 alters EC shape. Maximum intensity projections of confocal z-stacks of HUVECs transfected with dephospho-Marcksl1-EGFP (**a**, Marcksl1-AAA-EGFP) and phosphomimetic Marcksl1-EGFP (**b**, Marcksl1-DDD-EGFP) and stained with DAPI (blue) and phalloidin (red). Quantification of cell area (**c**), cell spikiness index (**d**) and cell aspect ratio (**e**; Marcksl1-AAA-EGFP: n=210 cells; Marcksl1-DDD-EGFP: n=200 cells). Analyzed by two-tailed unpaired *t* test. Violin plots represent the entire range of values, dotted lines indicate first and third quartiles, center line is median. Mean values are indicated. Scale bars, 25 μm. Source data are provided as a Source Data file.

a b

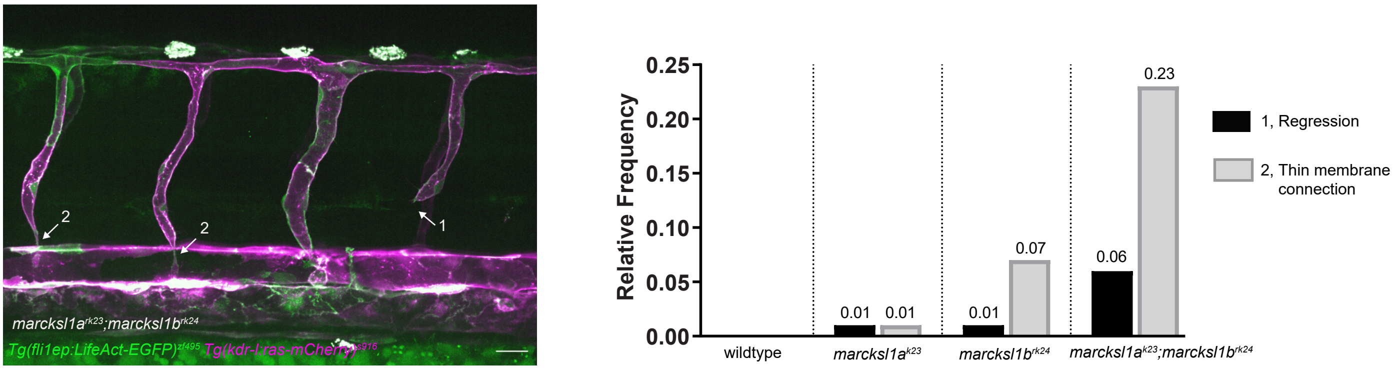

Supplementary Figure 12. Increased ISV regression in *marcksl1a*^{rk23}*;marcksl1b*^{rk24} embryos. **a** Maximum intensity projection of a confocal z-stack of ISVs in zebrafish trunk of 2 dpf *marcksl1a*^{rk23}*;marcksl1b*^{rk24} embryo in *Tg(fli1ep:Lifeact-EGFP)*^{zf495}*;Tg(kdr-l:ras-mCherry)*^{s916} background. Pheno-type (vessel regression and/or thin membrane connection) was observed in 20 out of 30 embryos. **b** Relative frequency of vessel phenotypes in wildtype and *marcksl1* mutant embryos (wildtype: *n*=45 ISVs/11 embryos; *marcksl1a*^{rk23}: *n*=116 ISVs/29 embryos; *marcksl1b*^{rk24}: *n*=100 ISVs/25 embryos; *marcksl1a*^{rk23}*;marcksl1b*^{rk24}: *n*=126 ISVs/30 embryos). Data collected from 2 independent crosses for every genotype. Scale bar, 20 μm. Source data are provided as a Source Data file.

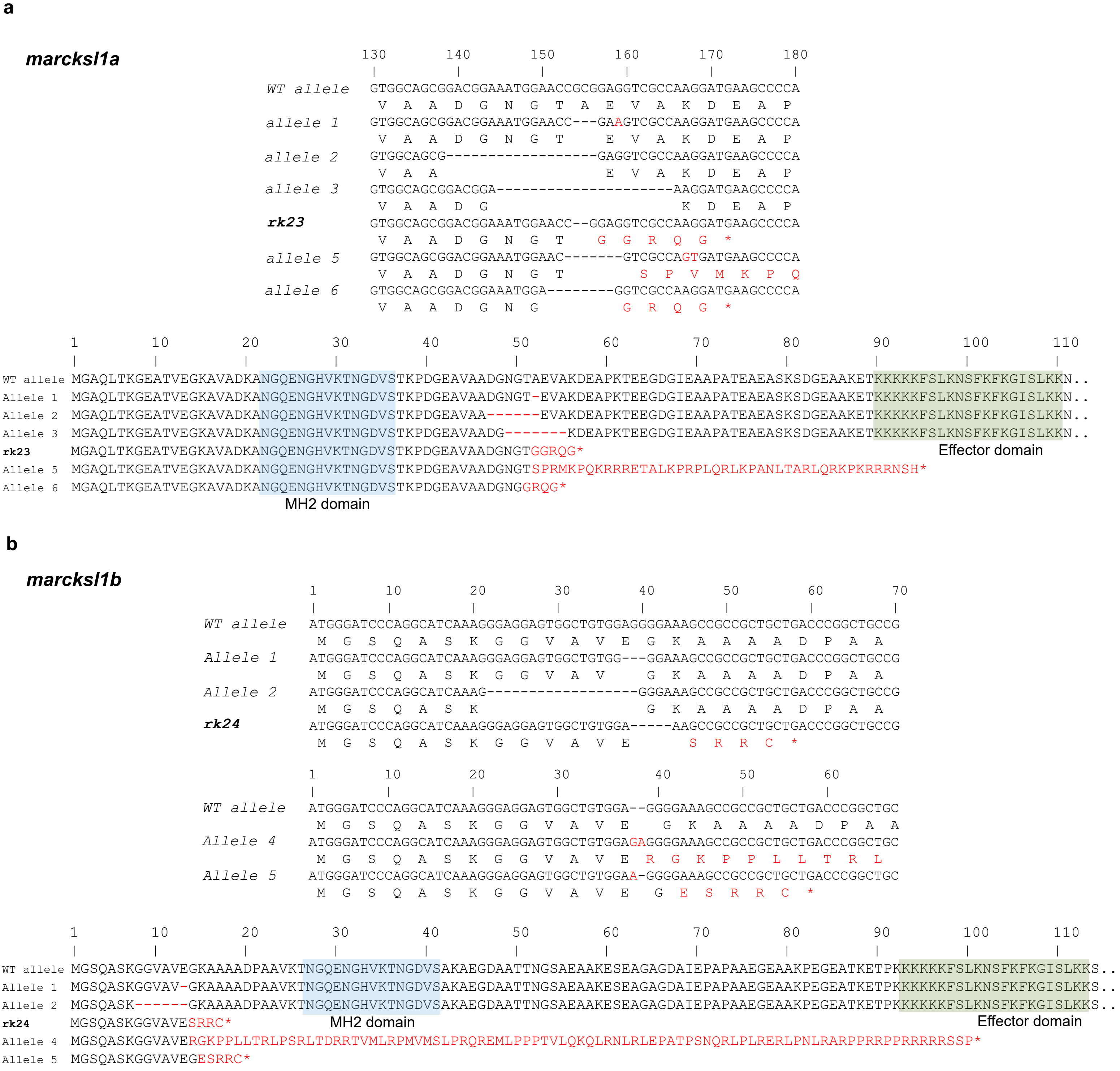

Supplementary Figure 13. *marcksl1a* and *marcksl1b* mutant alleles.

CRISPR/Cas9-induced mutations in *marcksl1a* gene and TALEN-induced mutations in *marcksl1b* gene. Partial genomic and protein (MH2 and Effector Domain are highlighted) sequences are shown. Missense amino acids and nucleotide substitutions/insertions are shown red.

Six *marcksl1a* mutants were obtained (**a**). Mutant alleles from 1 to 3 had a 3-nt, 18-nt and 21-nt deletions leading to 1, 6 and 7 amino acids deletions from protein, respectively, without affecting the open reading frame (ORF). Mutant allele 5 had a 7-nt deletion leading to a frameshift after T51 and premature stop codon at amino acid 94 after 43 missense amino acids. Mutant allele 6 had an 8-nt deletion leading to a frameshift after G50 and premature stop codon at amino acid 54 after 4 missense amino acids. Mutant allele *rk23* (used in all experiments) has a 2-nt deletion which leads to a frameshift after T51 and premature stop codon at amino acid 56 after 5 missense amino acids.

Five *marcksl1b* mutants were obtained (**b**). Mutant alleles 1 and 2 had a 3-nt and 18-nt deletions, respectively, leading to 1 and 6 amino acids deletions from protein without affecting the ORF. Mutant allele 4 had a 2-nt insertion leading to a frameshift after E13 and premature stop codon at amino acid 100 after 87 missense amino acids. Mutant allele 5 had a 1-nt insertion leading to a frameshift after G14 and premature stop codon at amino acid 19 after 5 missense amino acids. Mutant allele *rk24* (used in all experiments) has a 5-nt deletion which leads to a frameshift after E13 and premature stop codon at amino acid 17 after 4 missense amino acids.

Supplementary Table 1. Plasmids used in this study

Plasmid name	Plasmid backbone	Method of generation	Reference	Experiments	Comments
pMiniT-marcksl1a	pMiniT 2.0 (NEB)	PCR cloning	This paper	Suppl.Fig.1a	For riboprobe synthesis (1127 nt including 300 nt of 5'UTR and 185 nt of 3'UTR)
pMiniT-marcksl1b	pMiniT 2.0 (NEB)	PCR cloning	This paper	Suppl.Fig.1a	For riboprobe synthesis (1125 nt including 217 nt of 5'UTR and 284 nt of 3'UTR)
pMiniT-fscn1a	pMiniT 2.0 (NEB)	PCR cloning	This paper		Used to construct *6xUAS:fscn1a-T2A-mKate2CAAX* plasmid
**Expression constructs**					
fli1ep:marcksl1a-EGFP	pDestTol2CG2-*cmlc2:EGFP*	Gateway cloning	This paper	Suppl.Fig.2	Mosaic overexpression of wildtype Marcksl1a in ECs
fli1ep:marcksl1aΔED-EGFP	pDestTol2CG2-*cmlc2:EGFP*	Site-Directed mutagenesis	This paper	Suppl.Fig.2	Mosaic overexpression of mutated (without ED) Marcksl1a in ECs
fli1ep:marcksl1b-EGFP	pDestTol2CG2-*cmlc2:EGFP*	In-Fusion cloning	This paper	Suppl.Fig.2	Mosaic overexpression of wildtype Marcksl1b in ECs
fli1ep:marcksl1bΔED-EGFP	pDestTol2CG2-*cmlc2:EGFP*	Site-Directed mutagenesis	This paper	Suppl.Fig.2	Mosaic overexpression of mutated (without ED) Marcksl1b in ECs
6xUAS:marcksl1a-T2A-mKate2CAAX	pDestTol2CG2-*cry:mKate2*	In-Fusion cloning	This paper	Fig.2a-c, 5a,c	Mosaic overexpression of wildtype Marcksl1a in ECs
6xUAS:marcksl1aΔED-T2A-mKate2-CAAX	pDestTol2CG2-*cry:mKate2*	Site-Directed mutagenesis	This paper	Fig.2a-c	Mosaic overexpression of mutated (without ED) Marcksl1a in ECs
6xUAS:marcksl1aT124A-T2A-mKate2-CAAX	pDestTol2CG2-*cry:mKate2*	Site-Directed mutagenesis	This paper	Fig.2a-c	Mosaic overexpression of dephospho-Marcksl1a in ECs
6xUAS:marcksl1aT124D-T2A-mKate2-CAAX	pDestTol2CG2-*cry:mKate2*	Site-Directed mutagenesis	This paper	Fig.2a-c	Mosaic overexpression of phosphomimetic Marcksl1a in ECs
6xUAS:marcksl1b-T2A-mKate2CAAX	pDestTol2CG2-*cry:mKate2*	In-Fusion cloning	This paper	Fig.2d-f, 4a,c,d, 5b,d-f, 6, Suppl.Fig.5,8	Mosaic overexpression of wildtype Marcksl1b in ECs
6xUAS:marcksl1bΔED-T2A-mKate2-CAAX	pDestTol2CG2-*cry:mKate2*	Site-Directed mutagenesis	This paper	Fig.2d-f	Mosaic overexpression of mutated (without ED) Marcksl1b in ECs
6xUAS:marcksl1bT162A-T2A-mKate2-CAAX	pDestTol2CG2-*cry:mKate2*	Site-Directed mutagenesis	This paper	Fig.2d-f	Mosaic overexpression of dephospho-Marcksl1b in ECs
6xUAS:marcksl1bT162D-T2A-mKate2-CAAX	pDestTol2CG2-*cry:mKate2*	Site-Directed mutagenesis	This paper	Fig.2d-f	Mosaic overexpression of phosphomimetic Marcksl1b in ECs
6xUAS:fscn1a-T2A-mKate2CAAX	pDestTol2CG2-*cry:mKate2*	In-Fusion cloning	This paper	Fig.9a	Mosaic overexpression in ECs
fli1ep:lynEGFP	pDestTol2CG2-*cmlc2:EGFP*	Gateway cloning	This paper	Fig.4c,d	*In vivo* cell shape analysis (as a control)
fli1ep:Myl9b-EGFP	pDestTol2CG2-*cmlc2:EGFP*	Gateway cloning	This paper	Fig.1d-f, 6c, 8c	Used to generate *Tg(fli1ep:Myl9b-EGFP)* transgenic line
fli1ep:EGFP-PLCd1PH	pDestTol2CG2-*cmlc2:EGFP*	Gateway cloning	This paper	Fig.1a	Used to generate *Tg(fli1ep:EGFP-PLCd1PH)* transgenic line
pEGFP-N1			Clontech	Fig.4f-i, 8e-g	*In vitro* cell shape analysis and actin organization (as a control)
pEGFP-U6	pEGFP-N1		PMID: 18840295		Used to construct shRNA plasmids
**shRNA plasmids**					
pEGFP-CAAX-U6:shMARCKSL1<br>*pEGFP-CAAX-U6:shControl*	pEGFP-U6	Restriction/ligation cloning	This paper	MARCKSL1 KD (*in vitro* cell shape analysis and actin organization) Fig.4j-m, 8h-i	Original pEGFP-U6 vector was modified by in-frame fusion of the last 21 amino acids of human H-ras (CAAX box) to the C-terminus of EGFP, creating a membrane-targed EGFP.
**mouse Marcksl1 plasmids**					
pMarcksl1-EGFP	pEGFP-N1		PMID: 22751924	Fig.4f-i, 8d-g	*In vitro* cell shape analysis, actin organization
pMarcksl1-AAA-EGFP	pEGFP-N1		PMID: 22751924	Fig.8e-g, Suppl.Fig.11	*In vitro* cell shape analysis (dephospho-Marcksl1), actin organization
pMarcksl1-DDD-EGFP	pEGFP-N1		PMID: 22751924	Fig.8e-g, Suppl.Fig.11	*In vitro* cell shape analysis (phosphomimetic Marcksl1), actin organization
p5E-fli1ep			Gift from Nathan Lawson (Univ. Massachusetts Medical School)		Used as a source of *fli1ep* promotor
pDestTol2-cry:mKate2			Gift from Darren Gilmour (EMBL, Heidelberg)		Used as a backbone plasmid for In-Fusion cloning
pME-lynEGFP			Gift from Darren Gilmour (EMBL, Heidelberg)		To construct *fli1ep:lynEGFP* plasmid
pME-Myl9b			Gift from Holger Gerhardt (MDC, Berlin)		To construct *fli1ep:Myl9b-EGFP* plasmid
PLCd1PH			Gift from Carsten Schultz (EMBL, Heidelberg)		To construct *fli1ep:EGFP-PLCd1PH* plasmid

Supplementary Table 2. Oligonucleotides used in this study.

All synthetic oligonucleotides were purchased from Fasmac (Japan) and Invitrogen

**Primers for genotyping**	
Marcksl1a-Fwd	GTGTGTGTGTTTGCCAAGATGCATT
Marcksl1a-Rev	GGTTGCCACAGCGATATGAGATCAC
Marcksl1b-Fwd	AGCGCTGTAGGACTGGAACTGGTA
Marcksl1b-Rev	CAACGACAGAAATGAATCGAAAACG
**Primers for the full-length cDNA cloning**	
marcksl1a-fwd	ATGAAGCTCCAGCCCTCTTGTGCAGA
marcksl1a-rev	CGCGAACCAGTGAACGTTATCAGCA
marcksl1b-fwd	AGCGCTGTAGGACTGGAACTGGTA
marcksl1b-rev	TCCTCAACTCACTCTTGTGCTGACA
fscn1a-fwd	CATCATCCACGGTGCACCAGCAGA
fscn1b-rev	TGGCCACTCGTCAGGTCATCGA
**Primers for mutagenesis (deletion of an Effector domain)**	
marcksl1a-delED-fwd	GCAAGTGAGGAGGCAGCGGA
marcksl1a-delED-rev	GGTTTCCTTTGCAGCCTCGC
marcksl1b-delED-fwd	AATGCTGAGGTGAAGGAAGAGGC
marcksl1b-delED-rev	CTTGGGGGTCTCCTTGGTGG
**Primers for mutagenesis (T124A, T124D)**	
marcksl1aT124A-fwd	GGCTGTGGCCgcaCCCACCACCG
marcksl1aT124D-fwd	GGCTGTGGCCgacCCCACCACCGC
marcksl1aT124-rev	TCCGCTGCCTCCTCACTTGC
**Primers for mutagenesis (T162A, T162D)**	
marcksl1bT162A-fwd	CCCTGTTGAAgCCCCCAAGGC
marcksl1bT162D-fwd	CCCTGTTGAAgaCCCCAAGGCCGAGG
marcksl1bT162-rev	GCAGCGGGTGTCTCCTCG
**Oligos for human MARCKSL1 shRNA construction**	
shRNA sense	GTGTGAACGGAACAGATGATG CCTGACCCACATCATCTGTTCCGTTCACACTTTTTTG
shRNA antisense	AATTCAAAAAAGTGTGAACGGAACAGATGATGTGGGTCAGGGATCATCTGTTCCGTTCACAC
shRNA scrambled sense	GAGTCATGGGTCAGTTATATGCCTGACCCACATATAACTGACCCATGACTCTTTTTTG
shRNA srambled antisense	AATTCAAAAAAGAGTCATGGGTCAGTTATATGTGGGTCAGGCATATAACTGACCCATGACTC
**Oligos for marcksl1a sgRNA construction**	
tracrRNA	AAAAGCACCGACTCGGTGCCACTTTTTCAAGTTGATAACGGACTAGCCTTATTTTAACTTGCTATTTCTAGCTCTAAAAC
sgRNA marcksl1a	TAATACGACTCACTATAGGACGGAAATGGAACCGCGG GTTTTAGAGCTAGAAATAGCAAG
**qPCR primers**	
MARCKSL1-fwd	ATCATGGGCAGCCAGAGCT
MARCKSL1-rev	TGCCTCATCTGTTCCGTTCACAG
GAPDH-fwd	GCCACATCGCTCAGACACCAT
GAPDH-rev	TGAAGGGGTCATTGATGGCAACA

Supplementary reference

1. Bjorkblom, B. *et al.* c-Jun N-terminal kinase phosphorylation of MARCKSL1 determines actin stability and migration in neurons and in cancer cells. *Mol. Cell. Biol.* **32,** 3513–3526 (2012).
