# Supplementary material for: Marcksl1 modulates endothelial cell mechanoresponse to haemodynamic forces to control blood vessel shape and size
Source: Nat Commun. 2020 Oct 30;11:5476. doi: 10.1038/s41467-020-19308-5 (PMC7603353; doi:10.1038/s41467-020-19308-5)
Supplement: Supplementary file 3 — Description of Additional Supplementary Files [file 41467_2020_19308_MOESM3_ESM.pdf]

Description of Additional Supplementary Files

File Name: Supplementary Movie 1
Description: **Formation of ISVs and DLAV in wildtype and marcksl1ark23; marcksl1brk24 embryos.**
Live-imaging of wildtype and mutant embryos in Tg(fli1ep:Lifeact-EGFP)zf495;Tg(kdr-l:rasmCherry)s916 background. 00:00, hours:minutes post fertilization.

File Name: Supplementary Movie 2
Description: **Marcksl1 overexpression deregulates blood vessel diameter.** Left panel: time-lapse imaging of an ISV of a 2 dpf Tg(kdr-l:ras-mCherry)s916 embryo. Right panel: an ISV with mosaic Marcksl1b-EGFP overexpression (OE) at 2 dpf. Increased Marcksl1b level leads to fluctuation in vessel diameter as well as ectopic filopodia formation and blebbing. 00:00, hours:minutes.

File Name: Supplementary Movie 3
Description: **Ectopic Marcksl1a expression in ECs induces excessive filopodia formation and membrane blebbing during ISV lumenization.** ECs expressing Marcksl1a-T2A-mKate2CAAX are in magenta while wildtype ECs are green. Movie was taken from a 31 hpf Tg(fli1ep:Lifeact-EGFP)zf495 embryo. 00:00; hours:minutes.

File Name: Supplementary Movie 4
Description**: Ectopic Marcksl1b expression in ECs induces basal blebbing in perfused vessels.** ECs expressing Marcksl1b-EGFP are in magenta while wildtype ECs are in green. Movie was taken from a 54 hpf Tg(fli1:Lifeact-mCherry)ncv7 embryo. For wildtype EC membrane behaviour, refer to control in Supplementary Video 2. 00:00; hours:minutes.

File Name: Supplementary Movie 5
Description: **Marcksl1-induced blebs are filled with blood plasma.** Time-lapse imaging of DLAV of 2 dpf Tg(Tg(kdr-l:ras-mCherry)s916 embryo (wildtype) and embryo with endothelial overexpression of Marcksl1-EGFP. Lumen is labelled with DextranRhodamine (magenta) while endothelial plasma membrane is in green. Marcksl1-induced blebs are comprised of apical and basal membranes (green). 00:00; minutes:seconds.

File Name: Supplementary Movie 6
Description**: Actin dynamics in Marcksl1-induced blebs.** 2 dpf Tg(fli1:Lifeact-mCherry)ncv7 embryo with mosaic expression of Marcksl1b-EGFP. EC with increased Marcksl1b expression (magenta) exhibit increased filopodia formation, basal blebs and irregular cell shape. In most blebs, actin (green) reassembly around the bleb cortex precedes retraction (box A) while failure to do so leads to bleb persistence (box B). Refer to Supplementary Video 11 for actin dynamics in a control embryo. 00:00; minutes:seconds.

File Name: Supplementary Movie 7
Description: **Non-muscle myosin II dynamics in Marcksl1-induced blebs.** Wildtype: dynamics of Myl9b-EGFP (green) in an ISV of a 2 dpf Tg(fli1ep:myl9bEGFP)rk25;Tg(kdr-l:ras-mCherry)s916 control embryo. Endothelial membrane is in magenta. Marcksl1 overexpression: 2 dpf Tg(fli1ep:myl9b-

EGFP)rk25 embryo with mosaic overexpression of Marcksl1b-EGFP. Myl9b-EGFP (green) accumulates at the neck of the bleb and eventually reassembles at the front of the bleb (magenta) before retraction. 00:00; minutes:seconds.

File Name: Supplementary Movie 8
Description: **Supplementary movie 8. Local weakening of EC cortex induces local membrane blebbing.** Laser ablation of an arterial ISV was performed on a 3 dpf Tg(kdr-l:ras-mCherry)s916 embryo. *, site of ablation. Note that surrounding membranes not ablated by laser remain unperturbed. Time-lapse imaging at a single plane of the vessel was taken. 00:00:00, minutes:seconds:milliseconds.

File Name: Supplementary Movie 9
Description: **Short-term inhibition of actin polymerization induces membrane blebbing in perfused blood vessels.** 2 dpf Tg(kdr-l:ras-mCherry)s916 embryos were treated with 0.4% DMSO (left panel) or 0.3 µg/ml Latrunculin B (right panel) and imaged 10 minutes later. Magenta arrow, apical bleb. Black arrow, basal bleb. 00:00, hours:minutes.

File Name: Supplementary Movie 10
Description**: Decreased blood flow normalizes Marcksl1-induced blebbing.** 2 dpf Tg(fli1:Lifeact-mCherry)ncv7 embryo with mosaic expression of Marcksl1b-EGFP was treated with 1X tricaine for 2 hours, 4X tricaine for 4 hours, washed for 1 hour and then returned to 1X tricaine for 3 hours. Magenta, Marcksl1b-overexpressing cell; green, actin. 00:00, hours:minutes after start of each treatment.

File Name: Supplementary Movie 11
Description: C**ortical actomyosin network is highly dynamic.** Time-lapse imaging of a perfused ISV from a 2 dpf Tg(fli1ep:myl9bEGFP)rk25;Tg(fli1:Lifeact-mCherry)ncv7 embryo. The EC cortex is composed of a dynamic meshwork of actin (magenta, top panel) and myosin II (green, bottom panel). 00:00; minutes:seconds.

File Name: Supplementary Movie 12
Description: **Ectopic expression of Fascin1a in ECs induces bleb formation and vessel dilation in perfused blood vessels.** Time-lapse imaging of lumen formation in ISVs and DLAV of Tg(fli1ep:Lifeact-EGFP)zf495 embryo with mosaic expression of Fascin1a-T2A-mKate2CAAX from 30 hpf. Magenta, EC with Fascin1a overexpression; green, actin/wildtype ECs. 00:00; hours:minutes.

File Name: Supplementary Movie 13
Description: **Inhibition of Arp2/3-mediated branched actin formation leads to membrane blebbing and deregulation of vessel diameter.** 2 dpf Tg(kdr-l:ras-mCherry)s916 embryos were treated with 0.4% DMSO or 200µM CK666 for 1 hour and then imaged. 00:00, minutes:seconds.
