# Supplementary material for: Marcksl1 modulates endothelial cell mechanoresponse to haemodynamic forces to control blood vessel shape and size
Source: Nat Commun. 2020 Oct 30;11:5476. doi: 10.1038/s41467-020-19308-5 (PMC7603353; doi:10.1038/s41467-020-19308-5)
Supplement: Supplementary file 17 — Reporting Summary [file 41467_2020_19308_MOESM17_ESM.pdf]

nature research

Corresponding author(s): Li-Kun Phng

Last updated by author(s): Sep 15, 2020

Reporting Summary

Nature Research wishes to improve the reproducibility of the work that we publish. This form provides structure for consistency and transparency in reporting. For further information on Nature Research policies, see our Editorial Policies and the Editorial Policy Checklist.

Statistics

For all statistical analyses, confirm that the following items are present in the figure legend, table legend, main text, or Methods section.

n/a	Confirmed	
☐	☒	The exact sample size (*n*) for each experimental group/condition, given as a discrete number and unit of measurement
☐	☒	A statement on whether measurements were taken from distinct samples or whether the same sample was measured repeatedly
☐	☒	The statistical test(s) used AND whether they are one- or two-sided<br>*Only common tests should be described solely by name; describe more complex techniques in the Methods section.*
☒	☐	A description of all covariates tested
☒	☐	A description of any assumptions or corrections, such as tests of normality and adjustment for multiple comparisons
☐	☒	A full description of the statistical parameters including central tendency (e.g. means) or other basic estimates (e.g. regression coefficient) AND variation (e.g. standard deviation) or associated estimates of uncertainty (e.g. confidence intervals)
☐	☒	For null hypothesis testing, the test statistic (e.g. *F*, *t*, *r*) with confidence intervals, effect sizes, degrees of freedom and *P* value noted<br>*Give P values as exact values whenever suitable.*
☒	☐	For Bayesian analysis, information on the choice of priors and Markov chain Monte Carlo settings
☒	☐	For hierarchical and complex designs, identification of the appropriate level for tests and full reporting of outcomes
☒	☐	Estimates of effect sizes (e.g. Cohen's *d*, Pearson's *r*), indicating how they were calculated

Our web collection on statistics for biologists contains articles on many of the points above.

Software and code

Policy information about availability of computer code

Data collection	Software used for data collection are: Andor iQ (v3.6.3), Zeiss Zen 2 (black edition) and Leica LAS X (v3.0.1).
Data analysis	Software used for analyses are: Fiji (Image J v2.0.0-rc69/1.52n), RQ Manager (Applied Biosystems), Cell Ranger (10X Genomics, v2.1) and custom-made Python (v2.7) scripts that run on Fiji. Custom codes for in vivo cell shape analysis (https://github.com/dougkelly88/vessel_cell_shape_analysis), in vitro cell shape analysis (https://github.com/dougkelly88/HUVECShapeAnalysis) and analysis of membrane blebbing/actin and myosin II dynamics in blebs (https://github.com/dougkelly88/blebbing_analysis) have been deposited in GitHub. Custom code to analyse actin density and bundle width (ponden) can be obtained from https://dev.bioimageanalysis.jp/.<br>Plots were generated using GraphPad Prism (v 8.2.1).

For manuscripts utilizing custom algorithms or software that are central to the research but not yet described in published literature, software must be made available to editors and reviewers. We strongly encourage code deposition in a community repository (e.g. GitHub). See the Nature Research guidelines for submitting code & software for further information.

Data

Policy information about availability of data

All manuscripts must include a data availability statement. This statement should provide the following information, where applicable:

- Accession codes, unique identifiers, or web links for publicly available datasets
- A list of figures that have associated raw data
- A description of any restrictions on data availability

Data supporting the findings of this work are available within the paper and its Supplementary Information file. Any other data are available from the corresponding author upon reasonable request.

Field-specific reporting

Please select the one below that is the best fit for your research. If you are not sure, read the appropriate sections before making your selection.

☒ Life sciences ☐ Behavioural & social sciences ☐ Ecological, evolutionary & environmental sciences

For a reference copy of the document with all sections, see nature.com/documents/nr-reporting-summary-flat.pdf

Life sciences study design

All studies must disclose on these points even when the disclosure is negative.

Sample size	For zebrafish experiments, the sample size (number of cells or vessels) was chosen based on the number of embryos expressing the transgene of interest (e.g. transient overexpression of plasmids) or of the desired genotype obtained per experiment. The number of embryos used for time-lapse imaging per experiment ranged from 3 to 4 per experiment for overnight imaging, 6 to 8 for short (0.5 to 2 hours) but high temporal resolution imaging and 1 to 3 embryos for drug treatments since time was a limiting factor.<br>For cell culture experiments, the sample size (number of cells) was chosen based on the number of cells expressing the transgene of interest per experiment. For the analysis of cell shape, approximately 100 cells could be imaged per experiment by spinning disk confocal microscopy. However, due to the long duration that it takes to perform super-resolution imaging by Airyscan for the analysis of actin density, only 4 to 6 cells were imaged per experiment.
Data exclusions	No data was excluded from the analyses.
Replication	The number of independent experiments is specified in each figure legend, with at least 3 independent experiments unless otherwise stated.
Randomization	No method of randomisation was used as sample characteristic are defined by their unique genotypes.
Blinding	Investigators were not blinded during data collection and analysis. Blinding was technically difficult because experiments and analyses were done by the same investigators.

Reporting for specific materials, systems and methods

We require information from authors about some types of materials, experimental systems and methods used in many studies. Here, indicate whether each material, system or method listed is relevant to your study. If you are not sure if a list item applies to your research, read the appropriate section before selecting a response.

Materials & experimental systems

n/a	Involved in the study	
☐	☒	Antibodies
☐	☒	Eukaryotic cell lines
☒	☐	Palaeontology and archaeology
☐	☒	Animals and other organisms
☒	☐	Human research participants
☒	☐	Clinical data
☒	☐	Dual use research of concern

Methods

n/a	Involved in the study	
☒	☐	ChIP-seq
☒	☐	Flow cytometry
☒	☐	MRI-based neuroimaging

Antibodies

Antibodies used	Alexa Fluor 568 Phalloidin (1:1000, ThermoFisher Scientific, A12380), DAPI (1:1000, ThermoFisher Scientific, D1306), anti-VE-cadherin D87F2 antibody (1:100, Cell Signalling, 2500s); anti-phosphohistone H3 Ser10 (1:250, Merck Millipore, 06-570), goat anti-rabbit IgG(H+L) Alexa Fluor 488 (1:1000, ThermoFisher Scientific, A11008), Anti-Digoxigenin-AP (1:5000, Roche, 11093274910).
Validation	All commercial antibodies and stains were validated by the manufacturers.<br>- The anti-VE-cadherin antibody has been tested for specificity in HUVECs (Human Umbilical Vein Endothelial Cells) in immunofluorescence staining and does not cross-react with other cadherin family proteins e.g. N-cadherin. It is also used for western blotting, immunoprecipitation and flow cytometry. https://en.cellsignal.jp/products/primary-antibodies/ve-cadherin-d87f2-xp-rabbit-mab/2500?Ntk=Products&Ntt=2500<br>- The anti-phosphohistone H3 antibody has been validated in immunocytochemistry, western blotting and immunoprecipitation and has been demonstrated to react against several species including zebrafish. https://www.merckmillipore.com/JP/ja/product/Anti-phospho-Histone-H3-Ser10-Antibody-Mitosis-Marker,MM_NF-06-570?ReferrerURL=https%3A%2F%2Fwww.google.com%2F<br>- The anti-rabbit IgG(H+L) Alexa Fluor 488 antibody has been purified and cross-adsorbed against human IgG, human serum, mouse IgG, mouse serum and bovine serum to minimize cross-reactivity and increase sensitivity against rabbit IgG. This antibody is used for immunohistochemistry, immunofluorescence, western blotting etc in many publications. https://www.thermofisher.com/antibody/product/Goat-anti-Rabbit-IgG-H-L-Cross-Adsorbed-Secondary-Antibody-Polyclonal/A-11008<br>- The anti-digoxigenin-AP antibody has been used extensively in zebrafish in situ hybridisation protocols and is cited in Thisse and

Thisse, 2007.
- The Alexa Fluor 568 Phalloidin selectively stains F-actin and is used to visualize and quantitate F-actin in tissue sections, cell cultures and cell free-preparations. https://www.thermofisher.com/order/catalog/product/A12380#/A12380
- DAPI is a popular nuclear and chromosome counterstain for use in cells and tissues (Kubista et al., 1987).

References:
Kubista, M., Akerman, B. and Nordén, B. Characterization of interaction between DNA and 4'-6-diamidino-2-phenylindole by optical spectroscopy. Biochemistry (1987).
Thisse, C. and Thisse, B. High-resolution in situ hybridisation to whole-mount zebrafish embryos. Nature Protocols (2007). doi:10.1038/nprot.2007.514

Eukaryotic cell lines

Policy information about cell lines

Cell line source(s)	Human umbilical vein endothelial cells (HUVECs, C-2519A) and Human Pulmonary Artery Endothelial Cells (HPAECs) were obtained from Lonza.
Authentication	HUVECs have been authenticated by the manufacturer to express CD31 and CD105, which are endothelial cell markers (https://bioscience.lonza.com/lonza_bs/JP/en/Primary-and-Stem-Cells/p/000000000000184665/HUVEC-%E2%80%93-Human-Umbilical-Vein-Endothelial-Cells%2C-Pooled%2C-in-EGM-2#). HPAECs have been characterized by morphological observation through serial passage by manufacturer and stain positive for CD31, an endothelial marker (https://bioscience.lonza.com/lonza_bs/JP/en/Primary-and-Stem-Cells/p/000000000000184970/HPAEC-%E2%80%93-Human-Pulmonary-Artery-Endothelial-Cells).
Mycoplasma contamination	Cells have been tested negative for mycoplasma by the manufacturer.
Commonly misidentified lines (See ICLAC register)	No misidentified cell line was used in the study.

Animals and other organisms

Policy information about studies involving animals; ARRIVE guidelines recommended for reporting animal research

Laboratory animals	Male and female zebrafish (Danio rerio) between 3 months to 1.5 years of age were used in the study. Wildtype strain (AB) were used, along with the following transgenic and mutant lines: Tg(kdr-l:ras-mCherry)s916, Tg(fli1ep:Lifeact-EGFP)zf495, Tg(fli1:myr-EGFP)ncv2, Tg(fli1:myr-mCherry)ncv1, Tg(fli1:Lifeact-mCherry)ncv7, Tg(fli1:GAL4FF)ubs3, Tg(UAS:EGFP-UCHD)ubs18, Tg(kdrl:EGFP)s843, Tg(fli1ep:myl9b-EGFP)rk25, Tg(fli1ep:EGFP-PLC1dPH)rk26, TgBAC(pdgfrb:GFP)ncv22, marcksl1ark23 and marcksl1brk24.
Wild animals	The study did not involve wild animals.
Field-collected samples	The study did not involve samples collected from the field.
Ethics oversight	All animal experiments were approved by the Institutional Animal Care and Use Committee at RIKEN Kobe Branch (IACUC).

Note that full information on the approval of the study protocol must also be provided in the manuscript.
